# Incorporation of Epilepsy into Life Skills Education: Perceptions of Primary School Learners in Mpumalanga and Limpopo Province—A Qualitative Exploratory Study

**DOI:** 10.3390/children10030569

**Published:** 2023-03-17

**Authors:** Thendo Gertie Makhado, Rachel Tsakani Lebese, Maria Sonto Maputle

**Affiliations:** 1 Department of Advance Nursing Sciences, Faculty of Health Sciences, University of Venda, Thohoyandou 0950, South Africa; 2 Research Office, Faculty of Health Sciences, University of Venda, Thohoyandou 0950, South Africa

**Keywords:** learners, life skills, epilepsy, education, inclusion, perceptions

## Abstract

Providing education on epilepsy is crucial, as this helps individuals to acquire the necessary knowledge and skills to effectively manage seizures while also reducing the stigma and misconceptions surrounding the condition. The aim of this research was to examine how learners perceive the integration of epilepsy education into life skills training. A descriptive-exploratory design was utilized for the study. The research took place in the provinces of Limpopo and Mpumalanga, located in South Africa, where primary schools in chosen rural communities were selected for the research. Six focus group discussions were conducted with learners aged 9 to 14 years in grades 4 to 7. Each group was comprised of six learners, resulting in a total of 36 individuals who satisfied the inclusion criteria. Data were collected from August to November 2022. Semi-structured interviews were utilized to collect data until saturation was reached. The collected data were analyzed with the assistance of ATLAS.ti. The study’s results underscore the significance of incorporating epilepsy education into life skills curricula at the primary school level, as revealed by two prominent themes that emerged: first, the reasons provided by students for the integration of epilepsy education into life skills training, and second, the preferred teaching methodologies for epilepsy education within the life skills curriculum as identified by learners. Trustworthiness and ethical consideration were ensured. It is recommended that guidelines for epilepsy life skills should be developed to enhance the wellbeing and academic performance of learners with epilepsy in primary schools in Mpumalanga and Limpopo provinces.

## 1. Introduction

Around 50 million people worldwide are thought to have epilepsy, which is a brain condition that is quite common and is defined by the occurrence of seizures [1]. Epileptic seizures can persist for several minutes and are distinguished by sudden falls, blank expressions, or involuntary twitching motions. It has been documented that the highest rate of epilepsy is among children, especially in the first years of life until the age of 10 years [2]. This was further supported by a study conducted by Minardi et al. [3], which revealed that a high incidence of epilepsy occurs at the earlier stages of life, from birth to 12 years. According to Wilmshurst et al. [4], it seems that epilepsy is most prevalent during childhood, specifically in children aged 6 to 12 years residing in rural regions of Kenya, with an incidence rate of 187 per 100,000 per year. The authors also noted that the proportion of epilepsy cases is higher in African children compared to other regions. This is consistent with the findings from a study conducted in South Africa, which revealed that epilepsy is more common in Sub-Saharan Africa than in other continents [5]. In that study, Ackermann et al. [5] reported that approximately 51% of children in the study population had epilepsy. Although the prevalence of epilepsy in children varies across different countries and regions, it remains a significant public health concern worldwide. The World Health Organization estimated that approximately 10 million children worldwide have epilepsy, with an incidence rate of 50 per 100,000 children per year [6]. People living with epilepsy (PLWE) worldwide face different challenges because of the nature of the condition. Musekwa et al. [7], Makhado et al. [8], and Yeni et al. [9] reported that PLWE feel discriminated against and stigmatized, which is mainly caused by misconceptions about epilepsy.

The adverse impacts of stigmatization and discrimination against people living with epilepsy (PLWE) may consist of exclusion from social circles, reduced self-confidence, deteriorating mental health, reduced quality of life, exacerbation of seizures, a tendency to conceal their condition, and an increased likelihood of non-compliance [10]. This sentiment was supported by the World Health Organization [11], which stated that PLWE may not seek care because of a fear of being identified. This means that because epilepsy occurs mostly in the early years of life, children living with epilepsy may suffer the negative effects of stigmatization. These children living with epilepsy are school learners in different schools who may drop out of school to isolate themselves as a result of stigmatization and discrimination, as schools have been recognized as one of the main places of stigmatization [12]. Additionally, research has shown that students with epilepsy are leaving schools because they feel alienated [13]. Furthermore, epilepsy can negatively affect children’s quality of life, resulting in social isolation, stigmatization, and psychological distress [9]. Epilepsy can have a significant impact on children’s academic performance, quality of life, and social functioning, leading to academic failure and reduced educational and employment opportunities later in life [14]. Studies have shown that children with epilepsy are at increased risk of academic difficulties, such as lower academic achievement, decreased school attendance, and higher rates of grade retention and dropout [13,15,16].

The main cause of discrimination against and stigmatization of PLWE is a lack of knowledge regarding epilepsy [7,17,18]. Therefore, it is believed that education on epilepsy from the primary school level may decrease this stigma, and perhaps the number of learners living with epilepsy who drop out of school due to feeling discriminated against may also decrease. It is paramount that information on epilepsy be taught starting from the primary school level, because this is the age at which epilepsy is most prevalent [2]. This study is important, because it helps to advance knowledge and understanding in a particular field and can inform policy and practice. In the case of epilepsy education, there are currently no known studies published with a focus on teaching young learners about epilepsy in order to promote positive attitudes towards individuals living with epilepsy, which in turn can reduce stigma. This highlights the need for research in this area. The objective of the present research was to investigate how students perceive the integration of epilepsy education into life skills training, which can help inform the development of effective educational programs and policies aimed at reducing stigma and promoting positive attitudes towards people with epilepsy. This underscores the importance of this study in contributing to the existing body of knowledge on epilepsy education and its potential impact on reducing stigma and improving the lives of people with epilepsy.

## 2. Methods

### 2.1. Study Design

The study employed a descriptive-exploratory research design to investigate the perceptions of learners on the necessity of integrating epilepsy into life skills education.

### 2.2. Study Setting

The study was conducted in the South African provinces of Mpumalanga and Limpopo, which are located in the eastern and far northern regions of the country, respectively. Mpumalanga shares borders with Swaziland and Mozambique, and Limpopo shares borders with Zimbabwe, Botswana, and Mozambique. Despite the significant geographical distance between the two provinces, the researchers selected them because of the diverse population groups residing in these areas. As they are geographically and culturally related, these provinces have the most varied populations among all the provinces in South Africa. Although there has been an improvement in the accessibility of basic services in the Limpopo and Mpumalanga provinces, there are still noticeable differences between urban, peri-urban, and rural areas. The provinces have predominantly rural areas with limited healthcare services. Javier [19] reported that Mpumalanga province has an unemployment rate of 35.2%, and Limpopo province’s unemployment rate is at 30.4%. Overall, both provinces have a predominantly rural population and are characterized by significant disparities in access to healthcare and basic services, which can affect the socioeconomic status of people living there. Based on this data, the researcher identified this population as one of interest and selected the study sample from this population.

### 2.3. Population

The participants of this study were learners from the provinces of Limpopo and Mpumalanga. It is crucial to comprehend the viewpoints of these learners concerning the integration of epilepsy lessons into life skills education since they are the intended beneficiaries of such teaching. The goal of this research was to establish guidelines for incorporating epilepsy into life skills education; hence, the perspectives of these students hold significant importance.

### 2.4. Sampling

The present research, which forms part of the GladAfrica Epilepsy Research Project (GERP), was conducted in the pre-selected rural regions of Limpopo and Mpumalanga. The settlements were selected using purposive sampling to account for cultural diversity. In the province of Limpopo, rural areas were selected in Malavuwe/Nweli (VhaVenda), Mtititi (VaTsonga), Bochum, and Modjadjiskloof (Pedis). Clara and Acornhoek (VaTsonga), Jerusalem (Swati), and Kwaggafontein were the selected rural settlements in the province of Mpumalanga (Ndebele).

#### 2.4.1. Sampling of the Schools

The study intentionally selected all public primary schools situated in the chosen rural communities of Limpopo and Mpumalanga provinces in South Africa. Only schools that agreed to take part in the research were included in the study.

#### 2.4.2. Sampling for Learners

The study utilized purposive sampling to choose learners from grades 4 to 7 in public primary schools to participate, based on the judgment that these students have a better comprehension of the necessity of epilepsy education at this stage. The study targeted primary school students in grades 4 to 7, including those with physical disabilities, who attend public schools in the selected communities. The researchers assumed that learners within this age range would have a solid understanding of the study subject, and their ages ranged from 9 to 14 years. This approach aimed to ensure equal opportunities for all students, including those who repeated classes. However, only learners who provided their consent were considered eligible to participate.

#### 2.4.3. Sample Size

According to Botma et al. [20], at least six to eight participants are ideal for a focus group to discourage feelings of overcrowding and promote discussion of differences and similarities. The study involved six focus group discussions held in primary schools located in selected rural communities within Mpumalanga and Limpopo Province. In order to cover all cultural groups, a total of six focus groups were chosen. Each focus group comprised six learners from grades 4 to 7. The total number of learners who participated in this study was 36. All met the inclusion criteria, and the point at which data saturation was reached was established based on the number of focus group sessions conducted. 

### 2.5. Pretest

The purpose of pre-testing was to ensure that the open-ended questions in the study were clear and easily comprehensible and to assess the research’s feasibility, as recommended by Hurst [21]. The preliminary testing consisted of multiple stages, which included questioning five primary school students from different communities who were not part of the study to ensure that the questions were comprehensible. The time it took to answer the open-ended questions was observed to determine whether it was reasonable, and any challenging or confusing questions or terms were removed from the semi-structured interview guide. Furthermore, the range of responses for each question was evaluated to ensure that it was sufficient and could provide the required information. All questions were confirmed to have been answered, and adjustments were made to any necessary revisions. Any inaccuracies identified in the research process or instrument were corrected before the primary research was conducted.

### 2.6. Data Collection

The researcher chose to conduct focus group discussions with learners from selected rural communities’ primary schools located in Mpumalanga and Limpopo provinces, as this was a suitable method for obtaining multiple perspectives on the specific topic [22]. Data were collected from August to November 2022, and only learners who provided written consent, and whose parents also provided written assent, were included in the study. The focus group discussions were conducted in a quiet, empty classroom provided by the primary schools. During the break time between the focus group discussions, learners were given time to play games to refresh their minds. Prior to the focus group discussions, the researcher engaged in small talks to create a relaxed atmosphere for the participants [20].

The focus group discussions were directed using a semi-structured interview guide that contained sequenced open-ended questions [20]. The participants were informed about the study’s objectives, expectations, and the fact that they could withdraw from the study without any consequences before the discussions commenced. The interviewer presented all the questions in the interview guide, covering various topics such as the learners’ views on epilepsy, the significance of integrating epilepsy into life skills education, the crucial life skills aspects related to epilepsy, and the teaching techniques that could be employed while incorporating epilepsy into life skills education. The focus group discussions were flexible and allowed participants to move in the direction that they deemed important. The researcher took precautions to maintain focus on the subject in a non-threatening way, and participants were assured of group privacy. The researcher utilized techniques such as asking, clarifying, contemplating, and paraphrasing to encourage participants to speak freely and to provide in-depth descriptions of the phenomenon. Field notes were made for observations that could not be captured on audiotapes, such as non-verbal cues, interview settings, and subjective sensations.

### 2.7. Data Analysis

The researcher employed the notice-collect-think (NCT) analytical process to analyze the data, utilizing the ATLAS.ti software. The software was used as a tool to support the fundamental stages of the analytical process, rather than solely relying on it for analysis [23]. After identifying themes during the analysis, they were finalized and grouped into columns based on their similarity. To ensure the trustworthiness of the analysis, an independent co-coder was responsible for analyzing the raw data. The researcher and co-coder then engaged in an electronic meeting to compare their independently identified categories.

### 2.8. Measures to Ensure Trustworthiness

Ensuring trustworthiness is an essential consideration in qualitative explorational research. The measures to ensure trustworthiness that were followed in the current research were dependability, conformability, credibility, and transferability [24]. To enhance the credibility of the study, the researchers conducted in-depth interviews with participants until they reached data saturation [25]. To ensure dependability in the study, an audit trail was maintained, and all researchers’ notes, transcripts, and data were saved for future reference. Moreover, participants were provided with the personal and professional details of the researchers, so that they could contact them if they had any queries or needed further clarification [20].

Confirmability was guaranteed through the authors’ review of the study to ensure the accuracy of information presented based on collected data. To ensure replicability, a second expert in the same field reviewed and contrasted the data collected, and the researcher and the independent co-coder compared their independently developed codes to reach a consensus, as mentioned by Burns and Grove [24]. By providing a detailed description of the research design, data collection methods, and data analysis technique, the transferability of the study was ensured [20].

### 2.9. Ethical Considerations

After receiving approval from the Human and Clinical Trial Research Ethics Committee at the University of Venda for ethical considerations (SHS/19/PH/37/2101), the researchers received authorization to undertake the study from the Department of Basic Education in the provinces of Limpopo and Mpumalanga, as well as by the districts, circuits, and principals of the selected schools. Prior to the start of the interview sessions, informed written consent was obtained from the legal guardian/parents of each participant. Participants were provided with detailed information about the possible risks and benefits associated with the study and were asked for their consent before participating in the study. Legal guardians/parents and participants were made aware that they had the right to withdraw from the study at any time without facing any consequences. Throughout the study, anonymity and confidentiality were maintained, and participation was completely voluntary.

## 3. Results

In this study, two main themes and nine sub-themes were identified. The two main themes included learners’ reasons for the integration of epilepsy education into life skills training and the teaching methods that were recommended by learners for epilepsy in life skills education. A summary of each theme’s sub-themes is included in the description in Table 1.

### 3.1. Theme 1: Learners’ Reasons for the Integration of Epilepsy Education into Life Skills Training

The first theme that emerged from this study was the reasons for including epilepsy in life skills education that were given by the participants.

These reasons underscored the importance of incorporating epilepsy into life skills education.

The sub-themes that emerged are described below.

#### 3.1.1. Sub-Theme 1.1: Empowering Self and Others

The results of this study revealed that one of the reasons that epilepsy should be included in life skills education is the need to empower the self and others. Participants in this study elaborated that learning about epilepsy in schools during life skills education would help them to feel empowered with knowledge and to empower others in the community with regard to epilepsy. The following narratives emerged:

‘*So that we can know about the illness. And that even at home will be able to teach people about it*’.

(Participant C, Focus group 1)

‘*Because it might happen in your family and you can be able to tell them that we were taught about this and what it does*’.

(Participant B, Focus group 2)

‘*Because it happens that… Its important because if it happens at home, you’ll be able to tell your parents what you were taught and how they can deal with it*’.

( Participant F, Focus group 2)

‘*Because if someone has it we will be able to tell because we will have the knowledge that if it happens we will know it is the disease*’.

(Participant D, Focus group 3)

#### 3.1.2. Sub-Theme 1.2: Protecting Self and Others (Prevention of Epilepsy)

According to study participants, it is vital to prevent epilepsy, since participants indicated that were they to be informed about the condition, they would be able to know how to safeguard others as well as themselves. The following narratives emerged from this study:

‘*So that we can know the causes and how we can protect ourselves from it*’.

(Participant F, Focus group 1)

‘*So we can know the causes and what is supposed to be done when someone is in danger*’.

(Participant A, Focus group 1)

‘*I would love to know how to prevent it*’.

(Participant A, Focus group 3)

‘*We want to know if a person with epilepsy should go to school and play with others*’.

(Participant F, Focus group 6)

#### 3.1.3. Sub-Theme 1.3: Learning about the Causes and Treatment of Epilepsy and Ways to Help Others with Epilepsy

Interviewed participants also stated that the reason they would like epilepsy to be included in life skills education in primary schools is that this will help them to have knowledge regarding the causes and treatment of epilepsy, as well as knowledge on how to assist others with epilepsy when they have seizures. The following quotes emerged:

‘*Yes it is important so that we know exactly what is the cause of it and how can we assist other people to also have knowledge about it*’.

(Participant A, Focus group 4)

‘*So that when you see someone having a fit in the streets, you’re able to assist them*’.

(Participant E, Focus group 1)

‘*In the classroom, it’s important because when we’re taught about it and our friends have it, we can help them*’.

(Participant C, Focus group 2)

‘*When we are taught about epilepsy our friends that has it will feel safe when we are together because we will know how to care for them*’.

(Participant A, Focus group 6)

#### 3.1.4. Sub-Theme 1.4: Learning How to Accept Others with Epilepsy

One of the reasons explored by participants in this study was the capacity to recognize that others have a right to exist as their own distinct individuals. This indicates that participants recognize the importance of being taught about epilepsy in life skills, since this may enable them to accept epileptics for who they are. The following narratives emerged:

‘*People need to know what to do when it starts and to know the symptoms and it will decrease discrimination*’.

(Participant E, Focus group 1)

‘*It means we will grow up knowing what it is, how to assist a person with epilepsy. so we can make friends and always accommodate them because we know*’.

(Participant E, Focus group 4)

‘*We will be able to help others who may have it and also help their families to understand it. We will stop laughing at them because we will know what it is*’.

(Participant D, Focus group 5)

#### 3.1.5. Sub-Theme 1.5: Feeling Confident in Helping Others

Participants stated that they might not only acquire knowledge from epilepsy education, but they might also acquire skills for ways in which to help people living with epilepsy when they are having seizures. These results revealed that participants would be able to help others with confidence, because they would be educated on epilepsy. The narratives that emerged were as follows:

‘*Because we will know if someone falls for us how to assist and we will have the confidence to play with a person with epilepsy because we will know how to assist when they fall*’.

(Participant B, Focus group 4)

‘*We will know how to take care of a person with it in our class and bring her back to school because no one will laugh at her anymore because we will also have confidence of helping her if she falls*’.

(Participant C, Focus group 5)

### 3.2. Theme 2: Learners’ Recommended Teaching Methods for Epilepsy in Life Skills Education

There are different recommendations that participants in this study suggested as good ways of teaching epilepsy in life skills education. Participants recommended these teaching methods because they perceived them as the best possible ways to learn about epilepsy. This is important, as learners are the recipients of epilepsy education. The following sub-themes emerged: visual learning, learning through practical demonstrations, repetitive learning, kinesthetic learning, and other ways of learning about epilepsy.

#### 3.2.1. Sub-Theme 2.1: Visual Learning

Participants revealed that they might learn better if they were being taught through visual methods of teaching. The visual method of teaching is one in which teachers display pictures, and students watch videos of cartoons that display a person with epilepsy and the ways in which they can be assisted. Participants further stated that it might not be easy for them to forget the things that they saw. The following narratives emerged:

‘*I would want to see it being played like cartoons we watch so that we don’t forget*’.

(Participant C, Focus group 4)

‘*I would like to watch videos when people with epilepsy are assisted when they have fallen*’.

(Participant F, Focus group 6)

‘*I would like to watch it as a video so that I practice it in class with others, that way I will be condent that when someone falls, I will do what I saw on the video*’.

(Participant D, Focus group 5)

‘*Sometimes there should be books of pictures about epilepsy because we love pictures we will read every day. And teachers should give us chance of teaching one another*’.

(Participant B, Focus group 4)

#### 3.2.2. Sub-Theme 2.2: Kinesthetic Learning

Participants in this study advocated for kinesthetic learning because of its capacity to connect the learning process to physical activity. They continued by explaining that in order for students to learn about epilepsy properly, teachers should have them play act in a drama that is connected to the condition. This would help students to retain information on how to help epileptics and to be able to practice it when a situation arises. The following narratives emerged:

‘*The teacher have to teach us again and again and make us act the drama of assisting a person with epilepsy so that we are sure sure we can assist even when the teacher is not in class*’.

(Participant E, Focus group 4)

‘*After taking notes the teacher can make us sing it. Or create a song of epilepsy because we love music*’.

(Participant B: Focus group 5)

‘*Show us a person acting to have it and we will understand what it is like to have epilepsy*’.

(Participant F, Focus group 3)

#### 3.2.3. Sub-Theme 2.3: Learning through Practical Demonstrations

Participants also recommended that learning through practical demonstrations might be another way for them to understand this condition and for them to learn how to assist people living with epilepsy, especially when they have seizures. Furthermore, it was illustrated that when a teacher uses demonstrations, this will give students skills for how to apply the methods learned in real situations. Quotations that emerged were as follows:

‘*I want them to show an example and come and stand in front of us and show us what is happening and how we can help someone*’.

(Participant A, Focus group 3)

‘*I think they should also bring nurses in our schools to teach us this. So that teachers also can learn and teach us*’.

(Participant A, Focus group 4)

‘*ani when someone has epilepsy they bubble, that if they abuse bubble we show them how to treat them as a demonstration*’.

(Participant F, Focus group 3)

#### 3.2.4. Sub-Theme 2.4: Repetitive Learning

Repetition is one of the learning methods that participants suggested in this study, stating that it might be effective if they are taught about epilepsy every day and also questioned about what they have learned. The following narratives emerged:

‘*I would like to be taught every Monday to Friday when going to school and to be taught in the morning before we start with our classes*’.

(Participant F (enthusiastically), Focus group 2)

‘*There should be epilepsy lessons everyday so that we learn it every day*’.

(Participant D, Focus group 4)

‘*The teachers should teach us every day without getting tired. There should be a period for epilepsy*’.

(Participant B, Focus group 6)

## 4. Discussion

Epilepsy is known as a neurological condition that affects millions of people worldwide. It is a condition that is often surrounded by misconceptions and negative attitudes, particularly in developing countries [7]. Educating learners about epilepsy in primary schools through life skills education is crucial in promoting awareness, understanding, and acceptance of epilepsy in society. This study interviewed learners located in the provinces of Mpumalanga and Limpopo to explore their perceptions of the integration of epilepsy into the curriculum of life skills education, as they are the recipients of teaching.

The study found that learners recognized the need to teach epilepsy in life skills education and illustrated the reasons for which epilepsy should be incorporated into the curriculum. Empowerment was a key theme, as learners believed that with epilepsy education, they could empower others by sharing their knowledge. Similarly, a study conducted by Higgins [26] showed how epilepsy specialist nurses contributed to supporting and empowering people living with epilepsy. This means that one can only be capable of empowering others when they have received specialized knowledge in a specific area.

Furthermore, education on epilepsy can reduce stigma and discrimination and can help learners to accept others with epilepsy. The study also highlighted the importance of learners feeling confident in helping others and in protecting themselves and others from epilepsy. Similarly, O’Neill et al. [27] illustrated that being educated about onchocerciasis-associated epilepsy might reduce stigma and discrimination. It has been revealed that when learners are taught about epilepsy, this will help them to know the causes and treatment of the condition, as well as ways to help others with epilepsy; students learn how to accept others with epilepsy instead of discriminating against them [28]. Makhado et al. [28] further stated that when learners are knowledgeable about epilepsy, they will even assist others at home, leaving the whole community knowledgeable about epilepsy, which therefore decreases stigma related to epilepsy. The other issues that emerged from this study were protecting the self and others (i.e., prevention of epilepsy), learning how to accept others with epilepsy, and feeling confident to help others. These sub-themes are consistent with previous studies that suggest that education on epilepsy can empower individuals, promote awareness, understanding, and acceptance of epilepsy in society, and improve attitudes towards people with epilepsy [28,29,30].

The second theme that emerged from the current study pertained to learners’ recommendations for teaching epilepsy in life skills education. Participants articulated their thoughts on the best methods of teaching epilepsy that would be memorable and would enable them to assist individuals with epilepsy during seizures. Specifically, the participants recommended visual learning, kinesthetic learning, learning through practical demonstrations, and repetitive learning as the most effective teaching methods to be employed in life skills education when teaching on epilepsy. These recommendations align with previous research studies, which suggest that incorporating diverse learning styles into teaching can improve learning and retention [28,31]. The use of visual aids and practical demonstrations can assist learners in comprehending and recollecting information, and kinesthetic learning can facilitate learners’ ability to apply their knowledge in real-life scenarios.

## 5. Conclusions

To sum up, the results of this research underscore the significance of integrating epilepsy as a subject into life skills education within primary schools. Educating students about epilepsy can facilitate awareness, comprehension, and acceptance of epilepsy within society, and it can also enhance attitudes towards individuals with epilepsy. Additionally, the study provides suggestions for ways to teach epilepsy in life skills education, which can improve the learning and retention process. These conclusions can aid in the creation of educational programs aimed at raising awareness and understanding of epilepsy within society. The researchers recommend the development of a special epilepsy textbook for primary schools to promote epilepsy awareness and understanding in society. Creating a specialized textbook about epilepsy for primary schools might play a crucial role in enhancing the educational interventions that aim to promote epilepsy awareness and understanding. The textbook would provide accurate information about epilepsy, its causes, symptoms, treatment, and ways to support people with epilepsy. It might also include stories of people with epilepsy, highlighting their challenges, strengths, and achievements to reduce stigma and promote empathy and inclusiveness.

## Figures and Tables

**Table 1:** Themes and Sub-themes of the Results.

Theme	Sub-Theme
1. Learners’ reasons for the integration of epilepsy education into life skills training	1.1 Empowering self and others 1.2 Protecting self and others (prevention of epilepsy) 1.3 Learning about the causes and treatment of epilepsy, as well as ways to help others with epilepsy 1.4 Learning how to accept others with epilepsy 1.5 Feeling confident to help others
2. Learners’ recommended teaching methods for epilepsy in life skills education	1.1 Visual learning 1.2 Kinesthetic learning 1.3 Learning through practical demonstrations 1.4 Repetitive learning

## Data Availability

The sharing of data does not apply to this article, because no new data were created or examined for the study.
